# Measuring healthcare preparedness: an all-hazards approach

**DOI:** 10.1186/2045-4015-1-42

**Published:** 2012-10-25

**Authors:** David E Marcozzi, Nicole Lurie

**Affiliations:** 1 Office of the Assistant Secretary for Preparedness and Response, US Department of Health and Human Services, 395 E Street, SW, 10th Floor, Suite 1075, Washington, DC 20201, USA; 2 Assistant Secretary for Preparedness and Response, 200 Independence Avenue SW, 638G, Washington, DC 20201, USA

## Abstract

In a paper appearing in this issue, Adini, et al. describe a struggle familiar to many emergency planners—the challenge of planning for all scenarios. The authors contend that all-hazards, or capabilities-based planning, in which a set of core capabilities applicable to numerous types of events is developed, is a more efficient way to achieve general health care system emergency preparedness than scenario-based planning. Essentially, the core of what is necessary to plan for and respond to one kind of disaster (e.g. a biologic event) is also necessary for planning and responding to other types of disasters, allowing for improvements in planning and maximizing efficiencies. While Adini, et al. have advanced the science of health care emergency preparedness through their consideration of 490 measures to assess preparedness, a shorter set of validated preparedness measures would support the dual goals of accountability and improved outcomes and could provide the basis for determining which actions in the name of preparedness really matter.

## Commentary

Despite years of planning and billions of dollars spent on disaster preparedness and response activities worldwide, the science of preparedness is in its infancy. The empirical evidence for much of health emergency preparedness is scant. As a result, it is challenging to define what it means to be “fully prepared.” Collectively, the world has dealt with numerous disasters, but only a handful of nations have confronted many. This has made it challenging to convincingly link the structures and processes for health preparedness to outcomes, particularly mitigation of morbidity and mortality.

Israel, in part because it has confronted numerous mass casualty events, has been at the forefront of medical preparedness planning, and has developed sophisticated structures and processes for dealing with such medical emergencies. As described by Adini, et al. [1], Sarpy, et al. [2], and Einav, et al. [3], elements of this system include standard operating procedures, drills and exercises for most conceivable events, and measures to inform continuous improvement following each drill, exercise, and actual event. Given the state of the evidence, these authors have relied on a rigorous use of expert opinion to develop their measures; many of the experts involved have had frequent and direct response experience.

In a paper appearing in this issue, Adini et al. [4] describe a struggle familiar to many planners—the challenge of planning for all scenarios. They contend that all-hazards, or capabilities-based planning is a more efficient way to achieve general health care system preparedness than scenario-based planning. The authors describe a systematic investigation of the components of their preparedness system that impact hospital preparedness. The paper represents an important advance on several fronts. First, it describes a system of measurement which is consistently applied to assess and improve the preparedness of hospitals. Second, it uses thoughtful analytic methods to answer the question of whether an all-hazards approach is an appropriate methodology for preparedness. Supporting this, the authors found moderate to strong correlations between preparedness measures for various kinds of disasters- including mass casualty, toxicologic, and biological events. In other words, the core of what is necessary to plan for and respond to one kind of disaster (e.g. biologic event) assists with for planning and responding to other types of disasters. This finding allows for improvements and greater efficiencies in planning and time-consuming and expensive drills and exercises; an important consideration given the current fiscal climate in countries worldwide. Third, the authors found that SOPs, training and drills made more of a contribution to overall preparedness than equipment and preparedness knowledge of personnel- an important observation that facilitates effective resource allocation.

The authors’ findings are consistent with recent releases of a US Presidential Policy Directive [5], and related guidance from the US Department of Health and Human Services [6,7], which shift the focus of preparedness planning in the US toward an all-hazards, capabilities-based approach.

One challenge faced by most countries, including Israel and the US, is finding a parsimonious set of measures to assess and improve preparedness. While Adini, et al., initially considered 490 measures to assess preparedness, we recognize that countries and systems may not be capable of consistently deploying and analyzing such a large volume of measures. Interestingly, the findings by Adini, et al., support a shift to an all-hazards approach which, enables a reduction in the number of measures. Future work should examine the correlations between measures, and could make use of techniques such as factor analysis to identify a short set of measures, or even scales, that could serve as proxys for a longer, more complex measurement set.

This paper does not address another critical issue—measuring the preparedness of the public health system. As public health and medical care are so interdependent, we hope that similarly rigorous work regarding the public health system is ongoing.

Adini, et al., have advanced the measurement of health care emergency preparedness. With limited resources, the necessity to find commonalities of approach and efficiency in all we do, including measurement, is critical. In the end, a set of validated preparedness measures would support the dual goals of accountability and improved outcomes and could provide the basis for determining which actions in the name of preparedness really make a difference.

## Competing interests

The authors declare that they have no competing interests.

## Authors’ contributions

DM and NL jointly drafted, edited, and finalized this editorial. Both authors read and approved the final manuscript.

## Authors’ information

David Marcozzi, M.D., MHS-CL, FACEP, serves as the Director of the National Healthcare Preparedness Programs (NHPP) within the Office of the Assistant Secretary for Preparedness and Response at the U.S Department of Health and Human Services (HHS). The NHPP provides leadership, evaluation and funding through grants and cooperative agreements to States, territories, and eligible municipalities to improve United States healthcare preparedness and enhance community and health system preparedness for public health emergencies.

Nicole Lurie, M.D., M.S.P.H, is the Assistant Secretary for Preparedness and Response (ASPR) at the U.S. Department of Health and Human Services (HHS). As such, she serves as the Secretary’s principal advisor on matters related to bioterrorism and other public health emergencies. Her office is the lead agency for federal public health and medical preparedness and response, helping the nation prepare for, respond to, and recover from disasters.

## Commentary for

Adini B, Goldberg A, Cohen R, Laor D, Bar-Dayan Y. Evidence-based support for the all-hazards approach to emergency preparedness.

## Disclaimer

The views expressed are solely those of the authors and do not necessarily represent those of the US Government or the Department of Health and Human Services.
